# Is orthopaedics entering the age of generative AI?—A narrative review of current applications challenges and future directions

**DOI:** 10.1002/ksa.70145

**Published:** 2025-10-27

**Authors:** Felix C. Oettl, James A. Pruneski, Balint Zsidai, Yinan Yu, Ting Cong, Thomas Tischer, Michael T. Hirschmann, Kristian Samuelsson

**Affiliations:** ^1^ Department of Orthopedic Surgery Balgrist University Hospital, University of Zürich Zurich Switzerland; ^2^ Department of Orthopaedic Surgery Tripler Army Medical Center Honolulu Hawaii USA; ^3^ Sahlgrenska Sports Medicine Center Gothenburg Sweden; ^4^ Department of Orthopaedics, Institute of Clinical Sciences, Sahlgrenska Academy University of Gothenburg Gothenburg Sweden; ^5^ Department of Orthopedics Skåne University Hospital Malmö/Lund Sweden; ^6^ Department of Computer Science and Engineering Chalmers University of Technology Gothenburg Sweden; ^7^ Department of Orthopaedic Surgery University of Pittsburgh Pittsburgh Pennsylvania USA; ^8^ Department of Orthopaedic Surgery University Medicine Rostock Rostock Germany; ^9^ Department of Orthopaedic and Trauma Surgery Malteser Waldkrankenhaus Erlangen Erlangen Germany; ^10^ Department of Orthopaedic Surgery and Traumatology Kantonsspital Baselland Bruderholz Switzerland; ^11^ University of Basel Basel Switzerland; ^12^ Department for Orthopaedics Sahlgrenska University Hospital Mölndal Sweden

**Keywords:** artificial intelligence, generative AI, large language models, orthopaedic surgery, surgical planning

## Abstract

Artificial intelligence (AI) in medicine is undergoing a pivotal transformation, evolving from discriminative models that classify data to generative AI systems capable of creating novel content. Generative AI is a type of artificial intelligence that can learn from and mimic large amounts of data to create content such as text, images, music, videos, code, and more. The generative AI paradigm relies on advanced architectures, including large language models (LLMs), which are likely to redefine key processes in the practice of clinical medicine. The imaging‐ and procedure‐heavy specialty of orthopaedic surgery is uniquely positioned to benefit from innovations in spatial reasoning, biomechanical analysis, and procedural planning using generative AI. Key applications are rapidly emerging, like streamlining clinical workflows through automated documentation, the mediation of patient‐provider communication and enhanced interpretability of complex medical information. While an exciting field the current evidence base is quite limited. The continued integration of these technologies promises to enhance surgical precision, democratise access to advanced planning, and ultimately improve patient outcomes. However, realising this potential requires overcoming significant challenges related to the ‘black box’ nature of models, data bias, and evolving regulatory oversight. Rigorous clinical validation through prospective trials will be essential to ensure the safe, effective, and equitable implementation of generative AI in the future of orthopaedic care.

**Level of Evidence:**

Level V.

**Abbreviations**
AI: artificial intelligence
CNN: convolutional neural network
EHR: electronic health record
FDA: Food and Drug Administration
GAN: generative adversarial network
LLM: large language model
ML: machine learning
PACS: Picture Archiving and Communication Systems
PCCP: Predetermined Change Control Plan
SaMD: Software as a Medical Device

## INTRODUCTION

### The new wave of AI in healthcare

For several years, the integration of artificial intelligence (AI) into medicine has been characterised by *discriminative* models—powerful algorithms trained to classify data and predict outcomes [49, 69]. These systems have shown proficiency in tasks such as identifying pathologies on medical images or forecasting disease risk over time [47, 49]. The generative era of AI is characterised not only by new data analysis methods, but also by the active *creation of* data [4, 15, 55, 64, 70]. This shift, powered by advanced architectures like large language models (LLMs, trained on vast amounts of data to “understand” and generate human‐like text), generative adversarial networks (GANs, which use two competing neural networks to generate new, synthetic data that mimics a real dataset), and diffusion models (generating data by progressively adding noise to a sample and then learning to reverse the process), marks a transition from AI as a diagnostic assistant to an active collaborator in design, planning, and communication [14, 53, 64]. Recent use‐cases include the creation of synthetic medical images for research, and the automated synthesis of evidence‐based treatment protocols [31, 44, 62].

### Unique opportunities in orthopaedics

Generative AI provides unique opportunities to interpret complex patterns and solve challenges associated with three‐dimensional anatomy, biomechanics and surgical hardware implantation. The daily challenges faced by surgeons—from mentally reconstructing complex fracture patterns from radiographs to planning multi‐planar osteotomies and selecting appropriately sized implants—are fundamental problems of spatial reasoning and design. Sophisticated platforms have been developed for the rapid, automated templating of patient‐specific surgical plans from medical imaging data, including bone segmentation, anatomic landmark identification, and implant positioning [47, 48, 49]. This has culminated in recent milestones such as the first Food and Drug Administration (FDA)‐approved AI surgical guidance system capable of providing real‐time, intraoperative measurements without radiation exposure, fundamentally shifting surgical practice from ‘educated guesswork to data‐driven certainty’ [52].

### Scope and objectives of the review

The current narrative review aims to provide a comprehensive analysis of the generative era of medical AI and its specific applications within orthopaedics. We will begin by tracing the evolution from earlier AI models to the current generative landscape. The core of the manuscript is dedicated to detailing the key applications emerging in orthopaedic practice, from AI‐guided surgical planning and custom implant design platforms to the use of mixed reality for training and LLMs for clinical decision support. Subsequently, we will explore the technical considerations for implementation, review the emerging clinical evidence, and discuss the significant challenges and limitations that must be addressed. The objective of the current work is to provide orthopaedic surgeons, clinical researchers, and healthcare administrators with the foundational knowledge required to understand, critically evaluate, and ultimately harness the power of generative AI to deliver the next generation of musculoskeletal care.

## BACKGROUND AND CONTEXT

### The trajectory of AI in orthopaedics

The application of AI to orthopaedic surgery research and practice did not begin with the complex models seen today [48, 49]. The journey started with traditional machine learning (ML) algorithms, which were primarily used for risk stratification and outcome prediction [46, 49]. For instance, early models focused on tasks like predicting fracture risk based on bone mineral density and clinical risk factors [34, 43]. The field then advanced with the adoption of deep learning, particularly convolutional neural networks (CNNs), which further advanced medical imaging analysis [26, 38, 47, 54, 60].

### The foundational distinction: Discriminative versus generative AI

The critical evolution into the current era of medical AI lies in the distinction between two fundamental paradigms.

- *Discriminative AI*, which characterised the previous wave of innovation, is designed to *classify* or *predict* [49, 69]. It answers specific, closed‐ended questions based on input data. For an orthopaedic surgeon, its function is analogous to asking, ‘Is there a fracture in this X‐ray?' or ‘Does this patient have severe osteoarthritis?'
- *Generative AI*, in contrast, is designed to *create* novel content that statistically resembles its training data [4, 15, 55, 64, 70]. Instead of just classifying, it can synthesise new information. The surgeon's query can now be, ‘Generate a 3D model of this patient's comminuted tibia fracture’ or ‘Create a patient‐friendly summary of this operative plan.’ This shift from interpretation to creation is the hallmark of the generative era [12].

## UPCOMING APPLICATIONS OF GENERATIVE AI IN ORTHOPAEDICS

**Figure 1:**
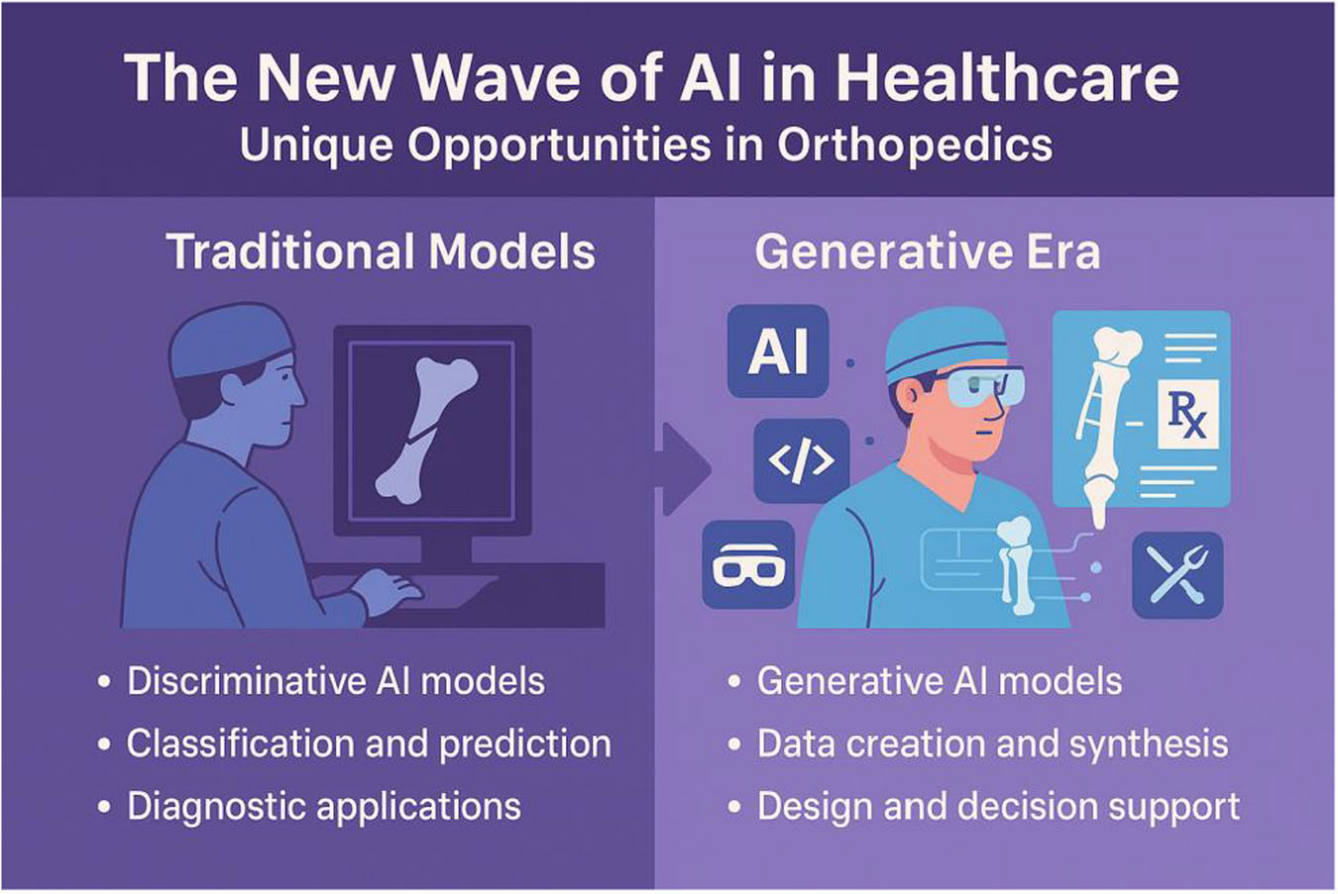
Changes in clinical practice due to generative artificial intelligence (AI). (This figure was generated with Chat GPT—for illustrative purpose.).

The integration of generative AI is actively reshaping multiple facets of orthopaedic care, from the operating room to the ward, the outpatient clinic and beyond. These tools are moving beyond simple data analysis to become active partners in creating patient‐specific solutions (Figure 1).

### Surgical planning and simulation

A cornerstone of modern orthopaedic surgery is precise preoperative planning. Generative AI assists in this domain by creating dynamic and interactive 3D models [21].

- Patient‐specific 3D anatomical modelling from CT or MRI scans. AI algorithms can analyse patient imaging to improve detailed 3D anatomical models [41]. This allows surgeons to gain a comprehensive understanding of a patient's unique anatomy before the procedure.
- Virtual surgical simulation to pre‐visualise outcomes, assess implant positioning as well as fit, and consequently anticipate intraoperative challenges. By integrating 3D models with virtual and augmented reality, surgeons can perform ‘virtual surgeries’ [35]. This enables them to rehearse procedures, such as knee arthroscopy, knee arthroplasty or fracture fixations, in a risk‐free environment.

### Custom implant and prosthetic design

Generative AI is shifting the paradigm from standardised 'one‐size‐fits‐all' implants to personalised devices engineered for an individual's specific anatomy and lifestyle [7, 22, 23, 28].

- *Generative design of patient‐matched implants (e.g., joint replacements, spinal cages and trauma plates) optimised for individual anatomy and biomechanics*. AI algorithms can analyse a patient's imaging data along with biomechanical parameters like weight and activity level to generate designs for implants that are a perfect match [28]. While patient specific implants and cutting guides have been on the market for some time, it was a time and resource intensive process which did not scale. AI can help with the creation of lighter, stronger, and more durable implants. Research from the University of Birmingham demonstrates the use of generative design to create bespoke High Tibial Osteotomy plates for osteoarthritis patients, showcasing a novel framework for load‐bearing, patient‐specific implants [28].
- *Exploration of novel lattice structures and materials to improve osseointegration and implant longevity.* By combining generative AI with 3D printing, it is possible to design implants with advanced features, such as intricate lattice structures that promote bone ingrowth [36]. Additionally, AI will be used to model and test new biocompatible materials and drug‐releasing frameworks to enhance healing and reduce inflammation [2, 27].

### Clinical decision support systems

The application of LLMs is streamlining clinical workflows and providing surgeons with data‐driven insights.

- *LLM‐powered ‘scribes’ for automated generation of clinical notes and operative reports*. LLMs are capable of summarising clinician's notes into coherent and readable clinical letters, reducing the administrative burden on medical professionals [20, 39, 58].
- *Agentic AI systems to assist in managing surgical workflows, from pre‐authorisation to scheduling follow‐ups*. Emerging agentic AI can automate complex tasks by interacting with different systems [5, 18, 30, 33, 44]. In orthopaedics, this could involve managing surgical schedules, handling pre‐authorisations, and monitoring postoperative recovery through wearable sensors, thereby improving efficiency, and enabling early detection of potential complications. Studies have shown that tailor made models like AMIE (Articulate Medical Intelligence Explorer) outperform human physicians on almost all metrics [62]

### Patient communication and informed consent

A significant barrier in healthcare is often the complex medical terminology used. Generative AI is helping to bridge this communication gap and promote a shift toward patient‐centred documentation and education, with the goal of improving patient understanding and compliance.

- *Use of LLMs to translate complex surgical plans and medical jargon into patient‐friendly language*. Studies have found that LLMs like ChatGPT can generate accurate and easy‐to‐understand educational materials for patients on topics such as total hip arthroplasty and osteoporosis [16, 17]. This potentially helps patients better comprehend their condition and treatment options [10, 40].
- *Generation of visual aids (e.g., personalised 3D models of a patient's own anatomy) to improve comprehension and facilitate shared decision‐making*. By providing patients with personalised 3D models of their own anatomy, surgeons can more effectively explain the planned surgical procedure. This visual approach improves patient understanding and fosters a more collaborative decision‐making process.

## TECHNICAL CONSIDERATIONS AND IMPLEMENTATION

Successfully integrating generative AI into orthopaedics requires careful planning around the underlying technology, data pipelines, and existing clinical systems. This section outlines the key technical pillars needed to support these advanced tools.

- *Data requirements*: The performance of Generative AI models hinges on access to large, high‐quality, and diverse datasets [29, 65]. In orthopaedics, this means curating vast repositories of multimodal information, including imaging studies (X‐rays, CTs and MRIs), electronic health record (EHR) data, and unstructured text from operative and clinical notes. The quality and variety of this training data are critical for building robust models that are both accurate and free from the biases associated with under‐representative data [29, 65].
- *Integration with clinical workflows*: For AI tools to be effective, they must integrate seamlessly into the existing hospital IT ecosystem. This presents a significant challenge, as it requires bridging the gap between AI platforms and legacy systems like Picture Archiving and Communication Systems (PACS) and EHRs. Strategies to overcome this involve using standardised data formats and developing middleware that allows different systems to communicate, ensuring that AI‐generated insights are available directly within the surgeon's established workflow without causing disruption. Meanwhile, Model Context Protocol aims to serve as a universal adapter for AI, standardising how AI models connect with various data sources and tools without requiring custom‐built integrations for each new application [25].
- *Validation and performance metrics*: Measuring the success of a generative AI tool is more complex than for traditional models. It requires a multi‐faceted approach to validation [6, 42]. For AI‐designed implants, success may be measured by their structural integrity and biomechanical performance. For generated 3D models, anatomical accuracy is paramount. In the case of synthesised clinical reports, the key metrics are clinical relevance and coherence, while being more cost efficient, which often require evaluation by human experts [61]. This ensures that the outputs are not just technically correct but are genuinely useful and safe for clinical application.

## CHALLENGES AND LIMITATIONS

Despite its transformative potential, the integration of generative AI into orthopaedic practice is not without obstacles. This highlights a critical gap between the technology's theoretical promise, often demonstrated in pre‐clinical studies, and its current state of validated, widespread clinical implementation. Addressing these technical, regulatory, ethical, and practical challenges is essential for the responsible and effective deployment of these powerful tools.

- *Technical hurdles*: A primary challenge is the “black box” nature of generative AI models, where the decision‐making process is not easily understandable to human users [8, 24, 45, 46, 57]. This lack of transparency is a significant hurdle in high‐stakes medical decisions and can erode trust among clinicians. For LLMs, the risk of ‘hallucination’—where the AI generates confident but factually incorrect information—poses a serious safety concern that necessitates rigorous human oversight [3, 13, 55, 67]. Furthermore, the significant computational expense required to train and deploy these sophisticated models represents a substantial financial and logistical barrier for many healthcare institutions.
- *Regulatory and approval pathways*: The dynamic, learning nature of generative AI presents a unique challenge for regulatory bodies. Traditional approval processes are designed for static devices, not software that can evolve over time. Furthermore, validating self‐improving models that can produce a vast range of outputs requires new testing methodologies beyond traditional verification. To address this, the FDA has introduced frameworks like the Predetermined Change Control Plan (PCCP), which allows developers to get pre‐approval for planned modifications to their AI algorithms, it is however unclear if that will be sufficient. Alongside the FDA's approach in the United States, European frameworks such as the EU AI Act and the Medical Device Regulation (MDR) are providing pathways that govern the approval and oversight of ‘Software as a Medical Device’ (SaMD) [19]. Navigating these evolving regulatory pathways for SaMD remains a complex and resource‐intensive process, though the FDA has cleared over 880 AI‐enabled devices, with a growing number in orthopaedics.
- *Ethical considerations and bias*: One risk associated with medical AI is the potential to perpetuate and even amplify existing biases in healthcare [9, 12, 30, 63]. If the data used to train a model is not diverse and representative of all patient populations, its outputs may be skewed. In orthopaedics, this could lead to AI‐designed implants that are not optimised for certain anatomies or treatment recommendations that are less effective for underrepresented demographic groups, thereby exacerbating health disparities. Key ethical concerns consistently raised include patient privacy, data security, informed consent, and accountability.
- *Integration and adoption barriers*: Overcoming cultural and practical barriers within the medical community is crucial for successful implementation. Studies show that while orthopaedic surgeons are optimistic about AI's potential, actual adoption in clinical practice remains in its early stages due to practitioner scepticism [56]. Many clinicians harbour concerns about the reliability of AI, the potential for overreliance to erode their own clinical judgement, and questions of who bears the legal liability in the event of an AI‐related error [32, 56]. Furthermore, the lack of clear reimbursement models for AI‐assisted care and the challenge of integrating these tools into existing hospital IT systems are significant hurdles to widespread adoption [56].

## FUTURE DIRECTIONS AND RESEARCH OPPORTUNITIES

The generative era of AI in orthopaedics is just beginning. The coming years will likely see the convergence of multiple technologies, leading to even more sophisticated applications that could fundamentally redefine standards of care. The following areas represent key frontiers for future innovation and research.

- *Personalised and automated rehabilitation*: Generative AI has the potential to revolutionise post‐operative care by creating personalised rehabilitation programs [1, 51]. Future systems could analyse real‐time data from wearable sensors tracking a patient's range of motion, activity levels, and gait [11, 50, 59, 66, 68]. Based on this continuous feedback, the AI could dynamically adjust physical therapy protocols, ensuring that each patient follows an optimal recovery path tailored to their specific progress and needs.
- *Robotics and intelligent automation*: The synergy between generative AI and robotics is one of the most exciting future directions. While AI is already being used for surgical planning, the next step is to integrate this intelligence more deeply with robotic systems that can execute those plans with superhuman precision and steadiness. This could lead to more automated surgical workflows, where the robotic system, guided by the AI‐generated plan, performs certain tasks autonomously while the surgeon supervises and manages the overall procedure. While not yet present in orthopaedics, researchers conducted ex vivo animal trials showcasing the capabilities of automation on the DaVinci platform [37]. This combination could minimise tissue damage, reduce operative time, and further enhance surgical accuracy.
- *Addressing identified research gaps*: For generative AI to achieve widespread, responsible adoption, the orthopaedic community must move beyond case studies and pre‐clinical validations. There is a critical and urgent need for multicenter consortia bundling resources and sharing data to responsibly develop useful clinical AI platforms. This approach is essential to rigorously validate the clinical efficacy, safety, and cost‐effectiveness of these emerging technologies in real‐world clinical practice. Future research must focus on generating robust evidence to definitively demonstrate that generative AI not only offers technical novelty but also delivers tangible improvements in patient outcomes and overall value to the healthcare system.

## CONCLUSION

In conclusion, the generative era of AI is beginning to reshape orthopaedics, offering powerful tools for personalising treatment, enhancing precision, and improving efficiency. To harness this potential, the orthopaedic community must adopt a strategy of critical evaluation and responsible innovation. Surgeons must engage with these tools while understanding their limitations; researchers must prioritise high‐quality clinical trials to build a robust evidence base; and healthcare leaders must develop strategic plans for adoption that address the complex technical, ethical, and financial challenges. By working collaboratively, the field can ensure that generative AI becomes a trusted, standard component of delivering the next generation of musculoskeletal care.

## AUTHOR CONTRIBUTIONS

All listed authors have contributed substantially to this work: Felix C. Oettl, James A. Pruneski and Balint Zsidai performed literature review, Felix C. Oettl performed primary manuscript preparation. Editing and final manuscript preparation was performed by James A. Pruneski, Balint Zsidai, Yinan Yu, Ting Cong, Michael T. Hirschmann and Kristian Samuelsson. All authors read and approved the final manuscript.

## CONFLICTS OF INTEREST STATEMENT

Kristian Samuelsson is a member of the Board of Directors of Getinge AB (publ) and medtech advisor to Carl Bennet AB. Ting Cong is a founder and board member of Sustain Surgical Inc. and Kondral Technology Inc. with no conflict to this work.

## ETHICS STATEMENT

The authors have nothing to report.

## Data Availability

Data sharing is not applicable to this article as no datasets were generated or analysed during the current study.
